# Surgically‐treated hemoptysis and alveolar hemorrhaging resulting from severe mitral regurgitation: A case report

**DOI:** 10.1002/ccr3.6924

**Published:** 2023-02-13

**Authors:** Daisuke Yamaguchi, Toshiya Tokui, Takahiro Narukawa, Masahiko Murakami, Tomotake Sekoguchi, Ryosai Inoue, Koji Hirano, Yasumi Maze, Hisato Ito

**Affiliations:** ^1^ Department of Cardiovascular and Thoracic Surgery Ise Red Cross Hospital Ise Japan; ^2^ Department of Internal Medicine Minamiise Municipal Hospital Minamiise Japan; ^3^ Department of Cardiovascular and Thoracic Surgery Mie University Hospital Tsu Japan

**Keywords:** alveolar hemorrhage, hemoptysis, mitral regurgitation

## Abstract

Cardiac etiologies of hemoptysis are less common. One such etiology includes mitral regurgitation. In patients with hemoptysis and unilateral consolidation, careful chest auscultation and cardiac assessment may assist in making an early diagnosis.

## Graphical abstract

This case suggests that MR can cause hemoptysis with unilateral pulmonary opacity. In patients with bloody sputum and unilateral consolidation, careful chest auscultation and cardiac assessment may lead to an early diagnosis.

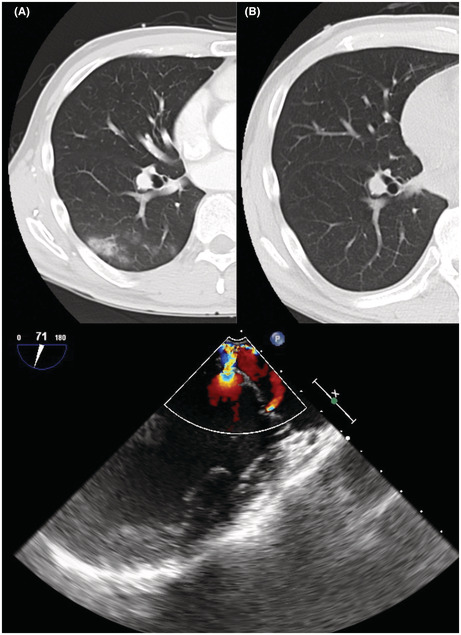

## 1 INTRODUCTION

Many pulmonary diseases can be complicated by hemoptysis, including tuberculosis, pneumonia, bronchiectasis, and malignant tumor.^1^ Cardiac etiologies are less common than pulmonary etiologies, and one particularly uncommon cardiac‐related cause of hemoptysis is mitral regurgitation (MR).

We herein report a case of hemoptysis and alveolar hemorrhaging caused by MR.

## 2 CASE REPORT

A 51‐year‐old man developed hemoptysis several years before we encountered him. He experienced weight loss of 10 kg while suffering from hemoptysis. One month before our encounter, he had a fever and visited a local clinic. Several infective diseases including COVID‐19 were suspected, but no evidence of those diseases was observed and COVID‐19 was denied by antigen testing. For a further examination, he was referred to the respiratory department of our hospital. His medical history included depression, and he was taking antidepressants. He had a brother under medical treatment for dilated cardiomyopathy.

On presentation, he was afebrile, and III/VI‐pansystolic murmur was best heard over the cardiac apex on chest auscultation. His leukocyte count and C‐reactive protein levels were not elevated.

**FIGURE 1:**
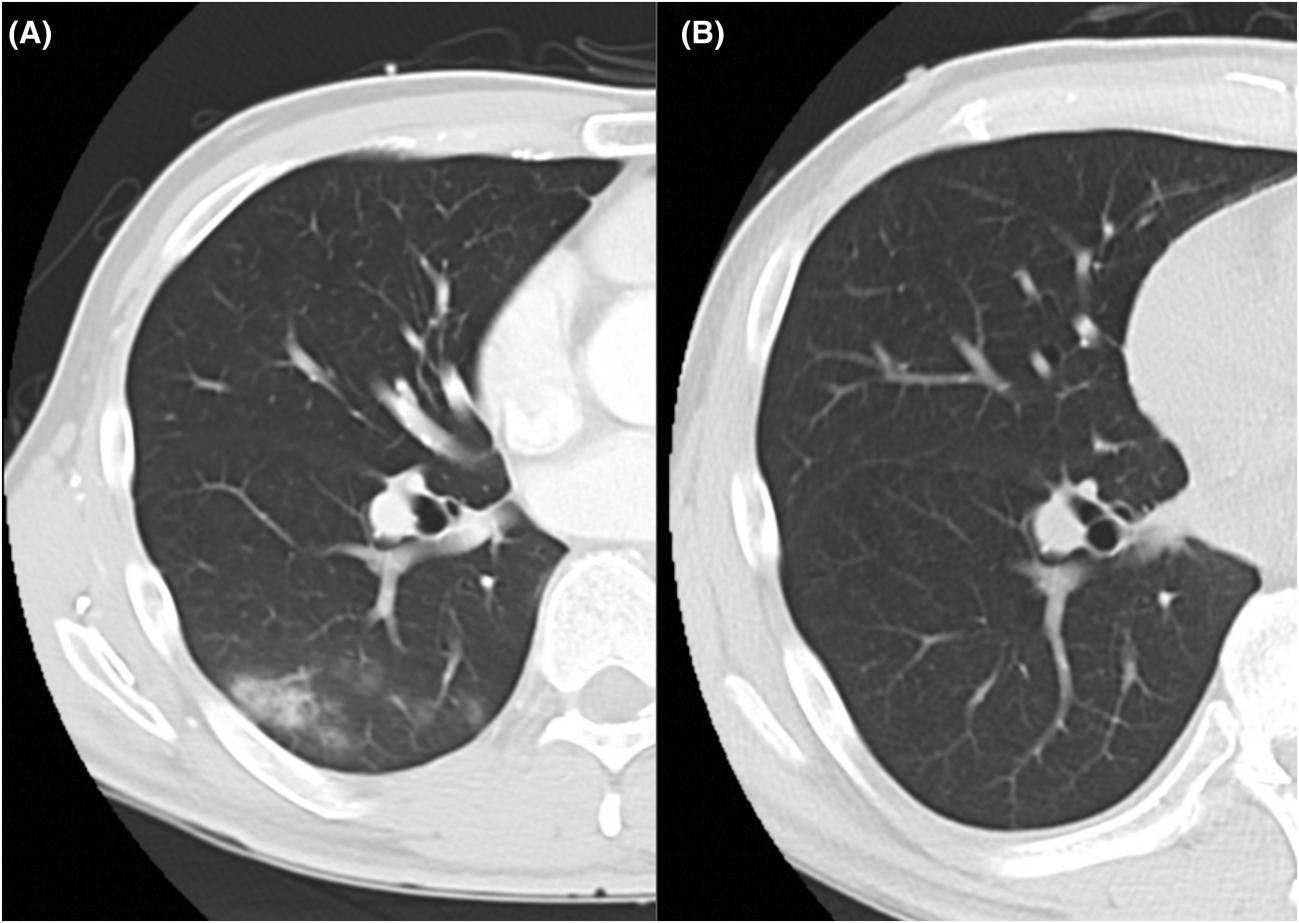
(A) Chest CT shows pulmonary opacity localized only in the superior segment of the right lower lobe. (B) On postoperative chest CT, no opacity was revealed.

**FIGURE 2:**
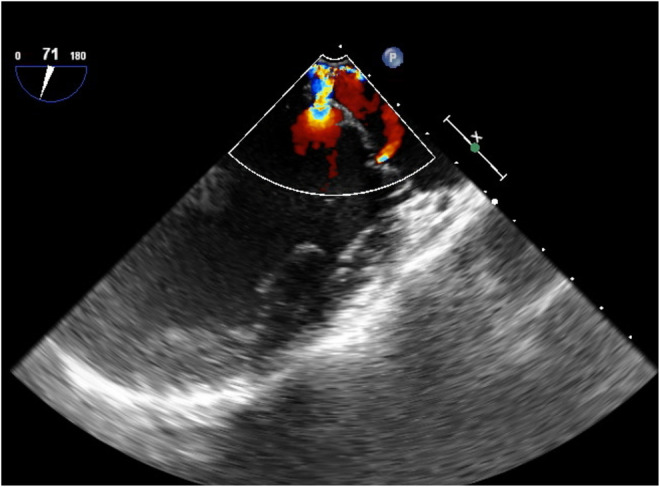
Representative images of transesophageal echocardiographic studies showing severe mitral valve regurgitation with ruptured mitral chordae tendineae.

Chest X‐ray revealed a normal cardio‐thoracic ratio, sharp costophrenic angle, and no congestion. Chest computed tomography (CT) showed pulmonary opacity localized only in the superior segment of right lower lobe and not detected on chest X‐ray (Figure 1A). Transthoracic and additional transesophageal echocardiograms (Figure 2) showed an ejection fraction of 58% with a normal ventricular size (left ventricular end‐systolic dimension 40 mm, left ventricular end‐diastolic dimension 58 mm) along with severe MR (Grade III) with prolapse of the mitral valve (A3). The regurgitant volume was 62 mL/beat, and the regurgitant fraction was 53%. No dilation of the left atrium was revealed. Mitral stenosis was not detected.

He was diagnosed with severe MR, and surgical intervention was indicated. Infection was denied, as he had no continuous fever, and his leukocyte count and C‐reactive protein levels were not elevated. There were no obvious symptoms or physical findings suggesting vasculitis or connective tissue diseases. The pulmonary infiltration was considered to suggest alveolar hemorrhaging. Because the left atrium was not dilated, the onset of MR seemed to be acute rather than chronic. We suspected that the cause of his bloody sputum was MR.

Preoperative pulmonary artery catheterization revealed a pulmonary artery pressure of 20/13 mmHg. An operation was performed with medial sternotomy, and cardiopulmonary bypass was established by ascending aortic and bicaval cannulation. After aortic cross‐clamping, left atriotomy was made under antegrade cardioplegic arrest, and the mitral valve was exposed. No vegetation was observed on either leaflet of the mitral valve. A2 prolapse was confirmed, and reconstruction of the ruptured chordae tendineae and annuloplasty with artificial valve annulus ring (Carpenter Edwards Physioring II 36 mm; Edwards Lifesciences) were performed. Left atrial appendage resection and a surgical cardiac biopsy were done simultaneously.

No evidence of cardiomyopathy was found in the biopsy specimen. Consolidation in the right lower lobe was not detected on postoperative CT (Figure 1B). After rate and rhythm control treatment for atrial fibrillation, he was discharged home on postoperative day 25 without recurrence of hemoptysis.

## 3 DISCUSSION

This case demonstrates three important points to discuss.

The first one is the relationship between alveolar hemorrhaging and MR. There are several possible reasons for the development of alveolar hemorrhaging in MR. The etiology of focal alveolar hemorrhaging is related to the reversal of flow in the right pulmonary veins during ventricular systole.^2^ The vector of the blood flow across an incompetent mitral valve may be selectively directed toward the right pulmonary venous system.^3^ Depending on the vector of the flow, the regurgitant blood flow would be directed toward the right pulmonary veins,^4^ resulting in numerous points of alveolar hemorrhaging and pulmonary edema. Factors affecting the distribution of edema include the volume of the regurgitant jet, the size of the left atrium, and the position of the pulmonary veins along the left atrial wall.^5^

The second point to discuss is the cause of hemoptysis. Most cases of bloody sputum result from pulmonary causes, such as bronchitis, bronchogenic carcinoma, and pneumonia.^1^ As a cause of hemoptysis, infection and lung cancer account for 60%–70% and 23%, respectively.^6^ Cardiac hemoptysis results from pulmonary venous hypertension due to left ventricular systolic heart failure,^6^ including mitral stenosis or regurgitation. Hemoptysis with an obvious murmur on auscultation is an early sign of a cardiac cause of hemoptysis, especially if there are no inflammation signs, such as a fever or leukocytosis. There is one possible reason for hemoptysis in acute mitral regurgitation: the hemodynamic state in acute mitral regurgitation changes more rapidly than in chronic mitral regurgitation. This results in a short time for the left atrium to adapt to the change and causes an increased left atrium pressure, resulting in alveolar hemorrhaging and hemoptysis.

The last point is the relationship between mitral regurgitation and pulmonary hypertension. In general, severe mitral regurgitation causes massive pulmonary hypertension and results in respiratory failure, which is sometimes severe enough to require mechanical cardiac support.^7^, ^8^ This is because an abrupt pressure elevation within the left atrium reflects back into the pulmonary circulation, leading to pulmonary hypertension and blood leakage from the capillaries.^9^ In contrast, in the present case, pulmonary artery catheterization revealed a normal pulmonary artery pressure despite severe mitral regurgitation. This may have been because MR did not affect the mean right ventricular afterload, as regurgitant jet flow was estimated to be limited in the right lower pulmonary vein. Only one study reported mitral regurgitation accompanied by a normal pulmonary pressure according to our search.^10^

The pulmonary opacity observed in the right superior segment in the present case may have been associated with the uneven distribution of regurgitant jet flow. Thanks to an appropriate diagnosis and treatment, our patient fully recovered from hemoptysis and avoided potential heart failure.

## 4 CONCLUSION

Our present case suggests that MR can cause hemoptysis with unilateral pulmonary opacity. In patients with bloody sputum and unilateral consolidation, careful chest auscultation and cardiac assessment may lead to an early diagnosis.

## AUTHOR CONTRIBUTIONS

DY prepared the manuscript. DY, KH, TS, and TT participated in the surgery. KH, YM, and TT supervised the writing of the manuscript. All the authors contributed to diagnosis and perioperative management of the patient, and read and approved the manuscript.

## FUNDING INFORMATION

No funding from any source was used for this study.

## CONFLICT OF INTEREST STATEMENT

All authors have no conflicts of interest to disclose.

## CONSENT

Written informed consent was obtained from the patient for publication of this report.

## Data Availability

Data sharing is not applicable to this article as no new data were created or analyzed in this study.
